# Precise Mechanical Oscillator Design and Calibration for Characterising Sub-Millimetre Movements in mmWave Radar Systems

**DOI:** 10.3390/s24237469

**Published:** 2024-11-22

**Authors:** Felipe Parralejo, Fernando J. Álvarez, José A. Paredes, Fernando J. Aranda, Teodoro Aguilera

**Affiliations:** 1 Sensory Systems Research Group (GISS), Department of Electrical Engineering, Electronics and Automation, Universidad de Extremadura, 06006 Badajoz, Spain; fafranco@unex.es (F.J.Á.); fer@unex.es (F.J.A.); teoaguibe@unex.es (T.A.); 2 School of Arts, Humanities and Social Sciences, University of Roehampton, Roehampton Lane, London SW15 5PU, UK; jose.paredes@roehampton.ac.uk

**Keywords:** configurable mechanical oscillator, mmWave radar, sub-millimetre movements

## Abstract

For many industrial and medical applications, measuring sub-millimetre movements has become crucial, for instance, for the precise guidance of surgical robots. The literature shows the feasibility of millimetre-wave (mmWave) radars to deal with such micro-vibrations. However, the availability of reference devices to configure and test these systems is very limited. This work proposes the design of a mechanical oscillator to characterise sub-millimetre vibration detection and measurement using a mmWave radar. The final implementation is fully controllable in both amplitude and frequency. Additionally, it can be wirelessly controlled and synchronised with other systems. Its functioning was experimentally calibrated and tested using the sub-millimetre motion capture system OptiTrack. It was tested to generate low-frequency oscillations from 0.80 Hz to 3.50 Hz with reliable peak amplitudes of 0.05 mm and above, with less than 6% peak amplitude relative error. Finally, the device was used to characterise a 60 GHz mmWave radar with those values.

## 1. Introduction

Measuring sub-millimetre movements is crucial for industrial applications, such as accurately moving hydraulic cylinders [1] or robot arms [2]. It is also very important in the medical field for the precise guidance of surgical robots for bone fracture alignment [3] or chest movement sensing for cardiopulmonary monitoring [4]. A lot of these challenges are solved with visual sensors [5] because of their well-known performance in tracking [6,7] and obstacle avoidance [8]. However, their precision might not be sufficient to reach the sub-millimetre level, and visible-light-based cameras present problems in low-light environments, in addition to the privacy issues that they can entail. Millimetre-wave (mmWave) radars are a potential alternative. These devices emit radiofrequency signals, so they can be used in dark or foggy environments [9]. Additionally, there are no privacy issues since they do not provide image-like information. Both features make them ideal for tasks such as high-precision pedestrian tracking in all-weather conditions for autonomous urban driving [10].

Nevertheless, this technology requires some tuning, which typically involves collecting large quantities of data. For instance, in [11], a calibration for a 60 GHz mmWave radar for close-range object location was performed through an RGB reference camera, obtaining a precision of a few millimetres. To reach sub-millimetre accuracy with this technology, researchers have used the intermediate-frequency signal’s phase, such as in [1], where a mmWave radar was installed on the piston of a hydraulic cylinder, similar to the proposals in [12,13]. This level of accuracy can also be leveraged to perform non-contact vital sign monitoring, such as in [14], where it is shown that, after a training phase, it is possible to measure sub-millimetre chest movements to estimate heart rate, or in [15], where a mmWave radar was used to detect human breathing in a non-line-of-sight region.

However, data collection procedures for tuning are time-consuming and require a large number of subjects to ensure that the proposed solutions are valid for everyone. In addition, the data must be diverse, be recorded at rest and include a high-intensity rate, which is physically hard to cover. For all these reasons, mmWave radars must be reliably configured, ensuring the correct detection of such sub-millimetre movements.

In medicine, dummy toys are used to train students how to auscultate or perform an RCP, but these devices are expensive and do not offer much control to the researchers, as they are intended for learning. They come with a set of heart and pulmonary sounds that students must learn to identify but do not produce significant movements that could be used for tuning. In physics laboratories, vibration generators are used to study harmonic oscillations. For example, the Vibration Generator no. 2185.00 by Frederiksen [16] is capable of producing vibrations of 0.1 Hz up to 5 kHz, which covers the range needed but with a fixed amplitude of 7 mm at 1 Hz that decreases with frequency and cannot be controlled, so it is not suitable for the task either. Some researchers have developed solutions based on their needs. For example, in [17], a left-ventricle simulator was built using a pump system to study different physiological and pathological conditions, and in [18], a bionic heart replica was made based on airflow to study heartbeats. In addition, in [19], a novel patient simulator was built to measure heart rate and arterial oxygen saturation using a pulse oximeter. Nevertheless, these solutions are unsuitable because they do not comply with the oscillatory requirements needed for the tests.

Thus, to the best of the authors’ knowledge, there is no commercial device capable of providing oscillatory movements that comply with the particularities of the application needed. There is a necessity for creating an ad hoc device that can be accessible to everyone, built with low-cost and easy-to-find components and able to create oscillations with the frequencies and amplitudes needed to serve as a testing tool for mmWave radars.

In this work, we propose a sub-millimetre mechanical oscillator system that complies with different amplitude and frequency requirements and is fully controllable via a socket server so that it can be controlled wirelessly. The design is based on a periodic movement whose frequency and peak amplitude can be controlled. So, it can be set to perform different oscillation patterns, and all the amplitude and frequency values, together with the timestamps of when they were commanded, are recorded for synchronisation with other devices. The system is built using low-cost components and 3D-printed parts, and the 3D model, the microcontroller C code and a script to wirelessly communicate with the device are provided in Appendix A. The accuracy of this device was tested and calibrated using the sub-millimetre Motion Capture OptiTrack system, which is theoretically capable of providing sub-20 μm accuracy [20]. Finally, this device was used to tune the configuration of the emission and reception parameters of a 60 GHz Frequency-Modulated Continuous-Wave (FMCW) mmWave radar to improve the accurate detection of sub-millimetre movements.

The rest of this work is structured as follows. In Section 2, the device’s design is presented, providing its limitations and operation principles. Later, in Section 3, the components of the system are listed, the precision tool used to calibrate the oscillator is described, and the mmWave radar sub-millimetre movement detection procedure is presented. Section 4 describes several calibration tests carried out for the oscillator to demonstrate the validity of the device. Next, in Section 5, the characterisation of oscillations using a mmWave radar is shown after proper configuration. And, finally, in Section 6, the main conclusions of this work are presented.

## 2. Design

**Figure 1:**
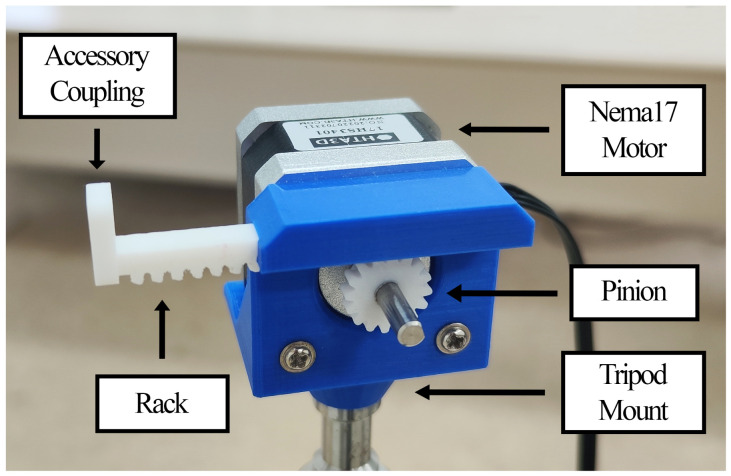
Proposed mechanical oscillator.

The proposed system, shown in Figure 1, comprises a Nema 17 stepper motor and a 3D-printed linear actuator that turns rotatory motion into linear movement. This actuator is composed of a pinion and a rack, which features an accessory coupling and a tripod mount. By means of an ATSAMW25 System on Chip (SoC), which contains an Arm Cortex-M0 32-bit SAMD21 microcontroller and an ATWINC1500 IEEE 802.11 b/g/n network controller, the amplitude and frequency of the linear movement can be wirelessly controlled.

Stepper motors are typically controlled using drivers like the DRV8825 [21] or the A4988 [22], which offer a simple interface with the motors and microstepping capabilities, increasing rotation resolution. This driver controls the motors by inputting Pulse Width Modulation (PWM) signals through two pins: DIR and STEP.

### 2.1. Basic Operation

Microcontrollers can generate PWM signals using internal timers. The SAM D21 microcontroller has four Timer/Counter for Control (TCC) application peripherals with different operation modes [23]. Typically, a dual-slope configuration is used when PWM signals are used to control motors. Using SAM D21’s dual-slope PWM bottom mode, TCCs count from ZERO to a predefined TOP value stored in the PER register and then back to ZERO. The output signal polarity is determined by the COUNT value, which is constantly compared to the value stored in the register CCx. When COUNT is below the value of CCx, the output is LOW, and it is HIGH at all other times. The number of counts needed to produce a period of the PWM signal is two times PER, and if $T_{\mathrm{CLK}}$ is the period of the clock that controls the timer, then the period of the PWM signal is

(1) $$T_{\mathrm{PWM}}=2\;\cdot \;\mathrm{PER}\;\cdot \;T_{\mathrm{CLK}}$$

$T_{\mathrm{CLK}}$ is the inverse of the CPU frequency of the SAM D21, $F_{\mathrm{CPU}}=48$ MHz, which can be scaled down using prescaler values of N={1,2,4,8,16,64,256,1024} to produce larger periods. Thus, the frequency of the PWM signal is

(2) $$f_{\mathrm{PWM}}=\frac{F_{\mathrm{CPU}}}{2\;\cdot \;N\;\cdot \;\mathrm{PER}}$$

The value of the register CCx controls the duty cycle of the signal. To produce a 50% duty cycle, the value of CCx must be half the value of PER.

### 2.2. Controlling Oscillation Frequency

**Figure 2:**
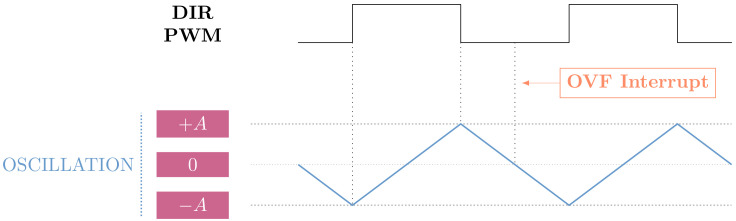
Oscillation generation using a PWM signal generated with the SAM D21 microcontroller.

A LOW value in the DIR pin of the motor driver sets the rotation counterclockwise, and a HIGH value clockwise. Thus, to produce an oscillation with a frequency *f*, the PWM signal inputted to the DIR pin must have a frequency $f_{\mathrm{PWM}}=f$ and a 50% duty cycle, as seen in Figure 2.

This frequency is limited by the maximum and minimum prescalers available for the CPU clock. The maximum frequency is half $F_{\mathrm{CPU}}$ using N=1 and PER=1. The minimum frequency is obtained with the maximum prescaler and PER values, N=1024 and $\mathrm{PER}=2^{24}$ (using 24-bit TCCs). Solving (2) with these values gives

$$\begin{array}{cc} f_{min} & =0.001397\;\mathrm{Hz}\\ f_{max} & =24\;\mathrm{MHz} \end{array}$$

### 2.3. Controlling Oscillation Amplitude

A LOW-to-HIGH transition on the STEP pin causes the movement of the motor in the direction set by the DIR pin. The magnitude of the movement is determined by the angular resolution of the stepper motor, given by the number of steps needed to complete a full revolution, but also by the microstepping setting of the driver. Each motor step is divided into multiple microsteps when enabled, increasing angular resolution. In the case of Nema 17 motors, 200 steps are required to turn $360^{\circ }$, resulting in a $1.8^{\circ }$ stepping angle. Using the maximum microstepping available to the DRV8825 driver, each step can be divided into 32 microsteps, giving an angular resolution of

(3) $$\theta_{\mathrm{res}}=0.05625^{\circ }/\mathrm{step}$$

If the desired angular amplitude is θ degrees, then the number of steps *N* needed to perform that movement is

(4) $$N=\frac{\theta }{\theta_{\mathrm{res}}}=\frac{\theta }{0.05625}$$

To produce an oscillation of peak amplitude *A* and period *T*, the number of steps given by (4) must be completed in time T/4. Thus, the duration of each step $t_{N}$ must be

(5) $$t_{N}=\frac{T/4}{N}$$

Every $t_{N}$ seconds, an impulse must be sent to the STEP pin of the motor driver. This can be accomplished using a 50% duty cycle PWM signal with a period equal to this duration.

**Figure 3:**
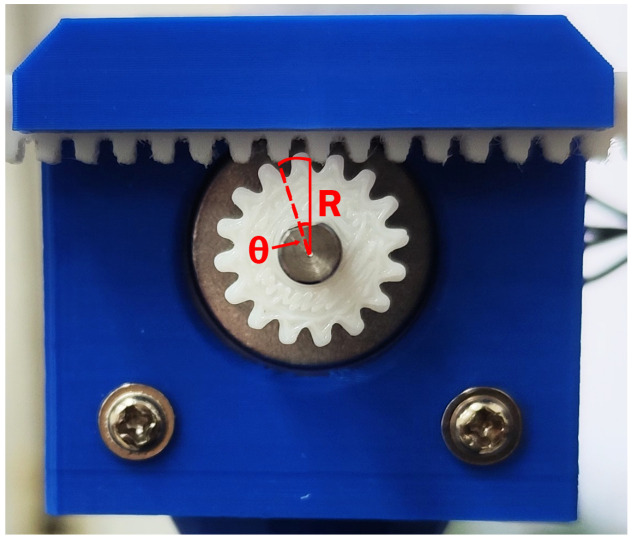
A 3D-printed linear actuator based on a rack-and-pinion drive system mounted on a custom-designed Nema 17 motor holder.

To turn rotatory motion into linear movement, a customised 3D-printed linear actuator has been designed based on the rack-and-pinion drive system shown in Figure 3. Because there is no reduction gear, the movement ratio is 1:1; thus, a gear rotation of θ degrees produces a linear movement equal to the arc of that angle, given by

(6) $$A=R\;\theta \;\frac{\pi }{180}$$

where *R* is the distance from the centre of the motor shaft to the point where the gear and the rack physically touch.

The angular resolution $\theta_{res}$ determines the minimum amplitude of the oscillation, whereas the maximum amplitude of the motion is determined by two factors. The first factor is the length of the rod connected to the gear and the distance from the edge of the motor holder to the centre of its shaft. And, the second factor is the maximum step frequency provided by the driver, but for the small amplitudes and frequencies desired, this is not a problem. In other words, in this case, the length of the rod is the most constraining part for the maximum amplitude. The limits for the designed actuator with R=7.45 mm are

$$\begin{array}{cc} A_{min} & =0.007314\;\mathrm{mm}\\ A_{max} & \approx 20\;\mathrm{mm} \end{array}$$

### 2.4. Updating Frequency and Amplitude in Real Time

When working with registers, it is possible to update their values in real time, thus producing a movement with variable frequency and amplitude. However, the equilibrium point of the oscillation must not be changed when performing these modifications. For this reason, all changes are performed when the overflow (OVF) interrupt triggers, which coincides with the moment the oscillation is at its equilibrium point. Looking at Figure 2, it is possible to see that this moment takes place when the amplitude of the oscillation reaches zero coming from +A. At this precise moment, the frequency or amplitude can be changed without perturbing the oscillating behaviour. It must be considered that this is possible because of the TCC operation mode used.

Using the ATWINC1500 network capabilities, the updates can be controlled wirelessly [24]. An access point is created when the system is turned on and waits for a user to connect. At that moment, the motor starts moving, and the user can change the movement frequency and amplitude at any time.

### 2.5. Oscillation Stabilisation

An important source of uncertainty in the real value of the amplitude is the number of steps given by (4). Let us consider an oscillation with the following characteristics:

$$\begin{array}{cc} f & =1.0\;\mathrm{Hz}\\ A & =0.5\;\mathrm{mm} \end{array}$$

**Figure 4:**
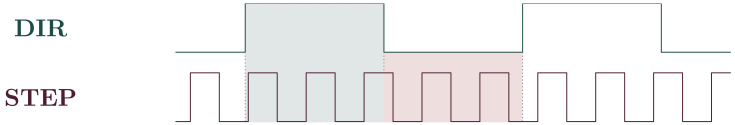
DIR and STEP PWM signals for some random amplitude chosen for representational purposes. Shadowed in green are the steps that take place during the positive direction movement, and in red are those during the negative.

**Figure 5:**
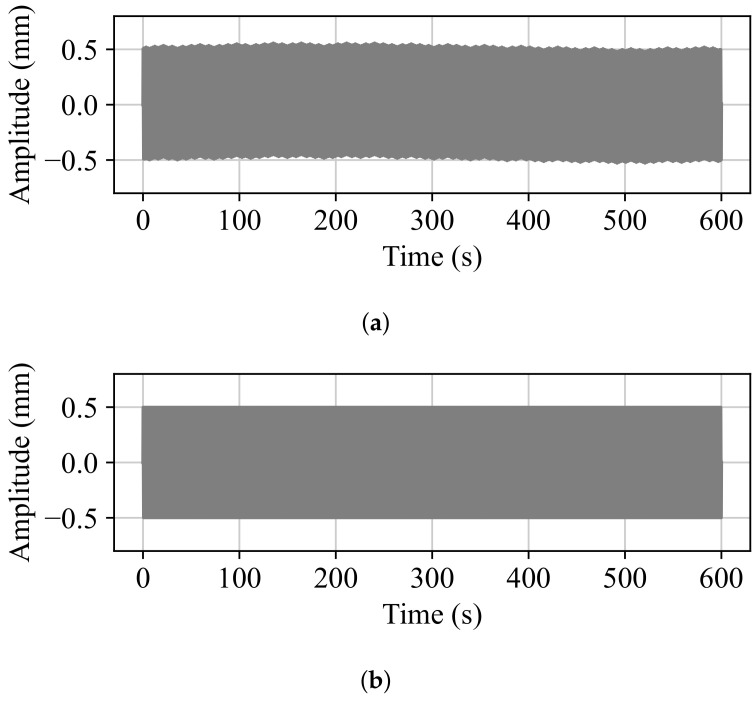
The amplitude evolution of a simulated 1 Hz oscillation with a 0.5 mm peak amplitude. (**a**) Instability because of an uncontrolled number of steps moved and *N* not being an integer. (**b**) Stability when the number of steps moved is controlled and equal to the integer part of *N*.

The number of steps given by (4) is N=68.3619. The effect that the number of steps’ decimal part has on the movement can be observed in the example in Figure 4. The number of LOW-to-HIGH transitions is inconsistent between the positive and negative directions of movement. In this case, for some periods of the oscillation, three steps will take place in one direction and only two in the other, but after some time, this pattern will be reversed, resulting in an unstable oscillation. This phenomenon can be seen in Figure 5a. To fix this, the decimal step must be discarded, losing up to one step in every T/4 period of the oscillation. However, because the movement direction is constant for T/2, except for the first T/4, the decimal part of 2N can be discarded. Thus, only a maximum of one step is lost in every direction of the movement, depending on the discarded decimal part, which results in a maximum absolute peak amplitude error of

$$\max\left\{A_{error}\right\}=A_{min}=0.007314\;\mathrm{mm}$$

and a stable oscillation, as seen in Figure 5b. To accomplish this, the STEP PWM signal period given by (5) is modified to be

(7) $$t_{N}=\frac{T/2}{\left\lfloor 2N\right\rfloor }$$

where ⌊·⌋ is the floor mathematical operator.

### 2.6. Other System Limitations

Apart from the physical limitations that set the maximum and minimum frequencies and amplitudes, the precision of the 3D-printed rack and pinion limits the accuracy of the amplitude. It is important that these parts always make proper contact so that the system does not fail to grip and the pinion moves without pushing the rack. These parts might have to be printed and checked several times to find the optimal grip.

## 3. Materials and Methods

This section will introduce the materials needed to build the mechanical oscillator as well as two commercial devices that are used: a precise sub-millimetre motion capture system to calibrate the oscillator and a mmWave radar to be evaluated using the calibrated oscillator.

### 3.1. Materials

The system is built with the following parts:

- ATSAMW25 SoC with a SAMD21 microcontroller and an ATWINC1500 network controller;
- A Nema17 17HS3401 stepper motor with a 1.8^∘^ stepping angle and 1.3 A rated current [25];
- A DRV8825 stepper motor driver with microstepping capabilities;
- A 100 μF capacitor;
- A 12 V, 2 A power supply.

**Figure 6:**
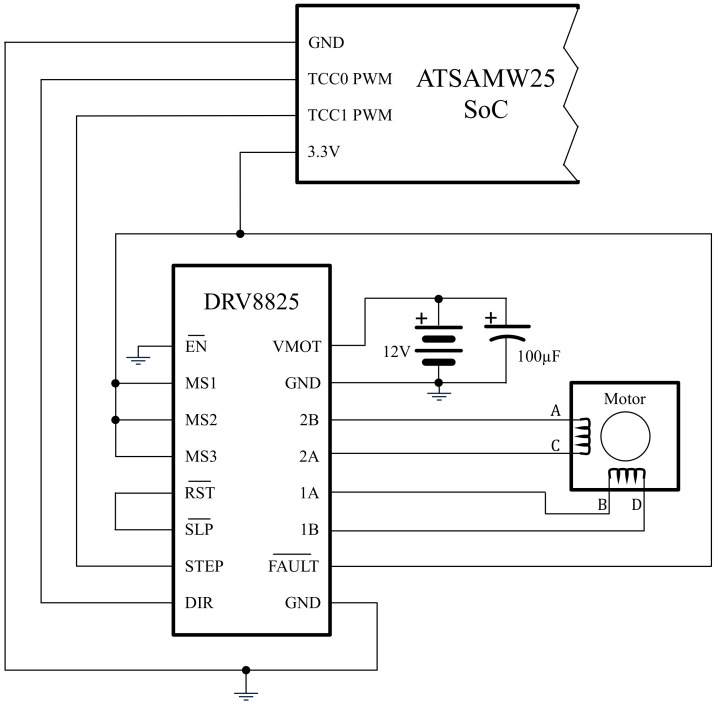
An electronic schematic of the designed system.

In addition, a digital oscilloscope is needed to check the PWM signals, and a multimeter is required to adjust the voltage reference (VREF) of the motor driver to control the current passed to the motor. For all the tests, this was set to 0.47 V (corresponding to approximately 200 mA). The schematic of the system is presented in Figure 6.

### 3.2. Precise Calibration System

**Figure 7:**
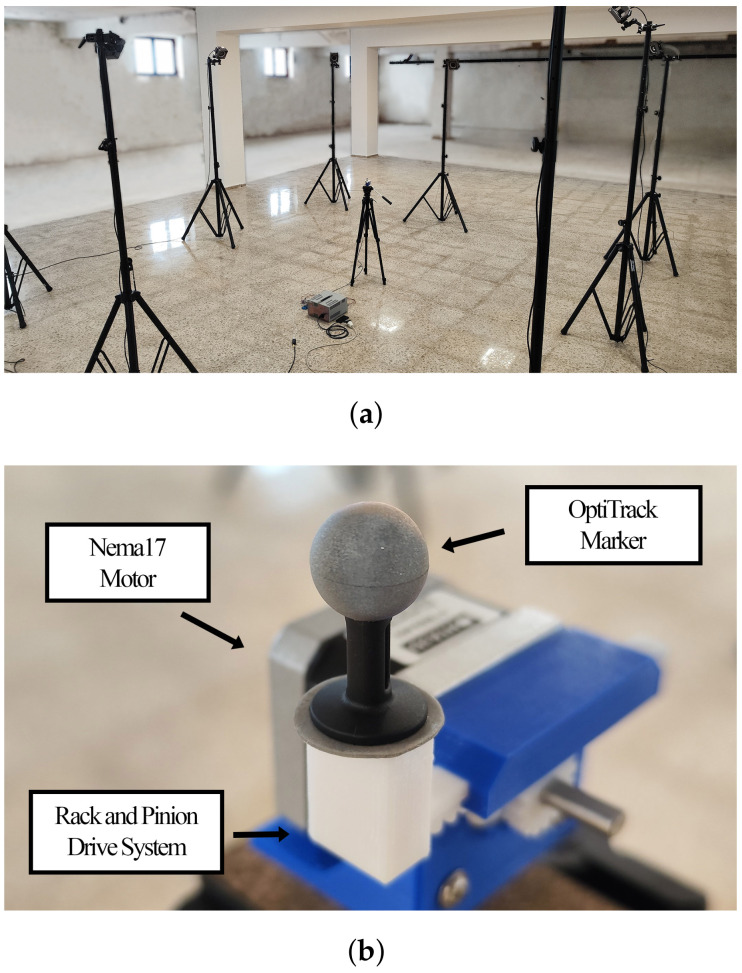
OptiTrack ground-truth system setup. (**a**) Mounted on a tripod at the centre is the Nema 17 motor, and on the ground are the electronics. OptiTrack cameras are mounted on tripods around them. (**b**) System close-up. The OptiTrack marker placed on the rack system.

The sub-millimetre motion capture system OptiTrack was chosen as a calibration tool to test and improve the functioning of the system. It was used to record the movement of the motor to study the amplitude and frequency of several oscillations, as described in Section 4. It was set up to cover an area of 2 × 2 m^2^, and the motor was mounted on a tripod and placed at its centre. The electronics and power supply were placed on the ground. This setup can be seen in Figure 7.

### 3.3. mmWave Radar Sub-Millimetre Accuracy

A mmWave radar device was employed to record the movements of the oscillator after it was calibrated to assess sub-millimetre oscillations measured with this technology.

As previously stated in Section 1, the phase of the intermediate-frequency (IF) signal is used for sub-millimetre precision. The IF signal is obtained after electronically mixing the transmitted and received signals and can be modelled as

(8) $$s_{IF}\left(t\right)=Ae^{j\left[2\pi f_{IF}t+\phi \left(t\right)\right]}$$

where *A* is the signal amplitude, $f_{IF}$ is the intermediate frequency and ϕ(t) is phase modulation due to target interaction (see [26] for more information).

In our case, we will have a single target with a range

(9) $$R\left(t\right)=R_{0}+x\left(t\right)$$

where $R_{0}$ is the target’s oscillation equilibrium point, and x(t) is the oscillation around it. The coarse range of the target $R_{0}$ can be extracted from the IF signal via a Fast Fourier Transform (FFT) because the intermediate frequency is related to the distance to the target as follows:

(10) $$f_{IF}=\frac{2BR_{0}}{cT_{r}}$$

where *B* is the radar’s effective bandwidth, *c* is the speed of light and $T_{r}$ is the ramp time. The sub-millimetre movement around $R_{0}$ is extracted for each frame from the phase of the FFT bin at that range. Then, the phase signal is obtained by concatenating the individual phases at different times *t*, giving

(11) $$\phi \left(t\right)=\frac{4\pi }{\lambda }\left(R_{0}+x\left(t\right)\right)$$

where λ is the mmWave radar signal’s wavelength.

A two-step procedure is followed to obtain the oscillation x(t) from (11). First, a 2D FFT is performed on the signal from different antennas to transform the frequency components of the IF signal into a range–azimuth heatmap, whose maximum indicates the target’s position. In the second part of the procedure, the raw phase is extracted from this maximum and unwrapped to account for phase changes larger than 2π. Finally, the mean of this signal is removed to isolate the oscillation x(t) from (11), resulting in the following relationship:

(12) $$x\left(t\right)=\frac{\lambda }{4\pi }\phi \left(t\right)$$

## 4. Calibration

This section describes several tests that were carried out to characterise the system, verify its operation and determine its accuracy limits using the precise sub-millimetre motion capture system OptiTrack.

As a matter of reference, we tested and calibrated the system according to heart rate, as this constitutes a challenge in the sub-millimetre-movement field. Our approach is substantiated by the fact that chest movement due to heart rate can be modelled using a simplified sinusoidal wave model [27], so our system produces a periodic movement within the peak amplitudes and frequencies corresponding to heart rate. Its frequencies range from slightly below 1 Hz [28] to ∼3.5 Hz [29]. The peak amplitudes vary depending on the individual, ranging from 0.2 to 0.5 mm [30].

### 4.1. Amplitude Calibration

**Figure 8:**
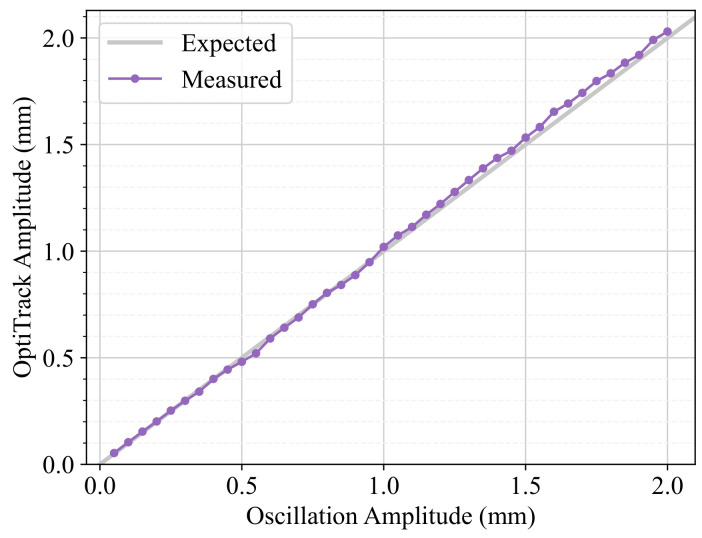
Amplitude calibration results. The amplitude measured using OptiTrack is plotted against the amplitude set in the device.

Because of minimal imperfections in the rack-and-pinion drive system, the resulting amplitude might not be exactly the one set in the parameters of the oscillation, but this can be corrected. To account for this calibration, first, a sweep of peak amplitudes was performed, and then the regions that needed calibration were adjusted using least-squares fitting. We studied the range A∈[0.05,2.00] mm in increments of 0.05 mm for an oscillation frequency of f=1.0 Hz and a collection time of 10 s. After performing minor adjustments, the study was repeated, resulting in the calibrated data seen in Figure 8, which shows that a good calibration was achieved. For all amplitudes, the relative error is lower than 6 %, resulting in a mean absolute error (MAE) of 0.02 mm, in agreement with the maximum error expected in Section 2.5.

### 4.2. Frequency Test

**Figure 9:**
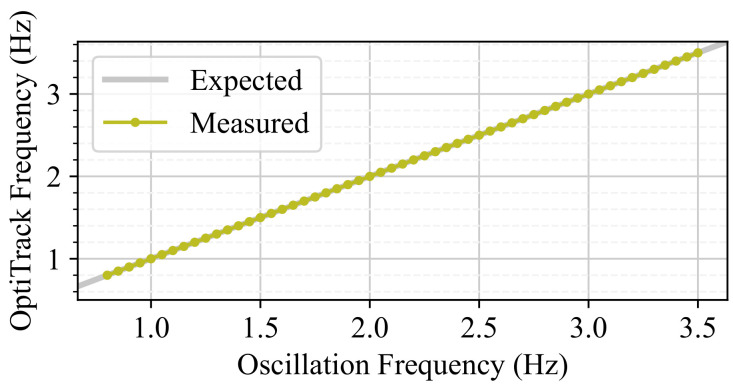
Frequency test results. The frequency measured using OptiTrack is plotted against the frequency set in the device.

For a fixed amplitude of A=1.0 mm, a sweep of frequencies was performed for values in the range f∈[0.80,3.50] Hz in increments of 0.05 Hz, with a collection time of 10 s. A spectral analysis was performed using an FFT to estimate the frequency component of the collected data, and the results are presented in Figure 9. The results show that the frequency of the oscillation is very accurate, with a 0.02 % relative error computed using four decimal places.

### 4.3. Amplitude Change

**Figure 10:**
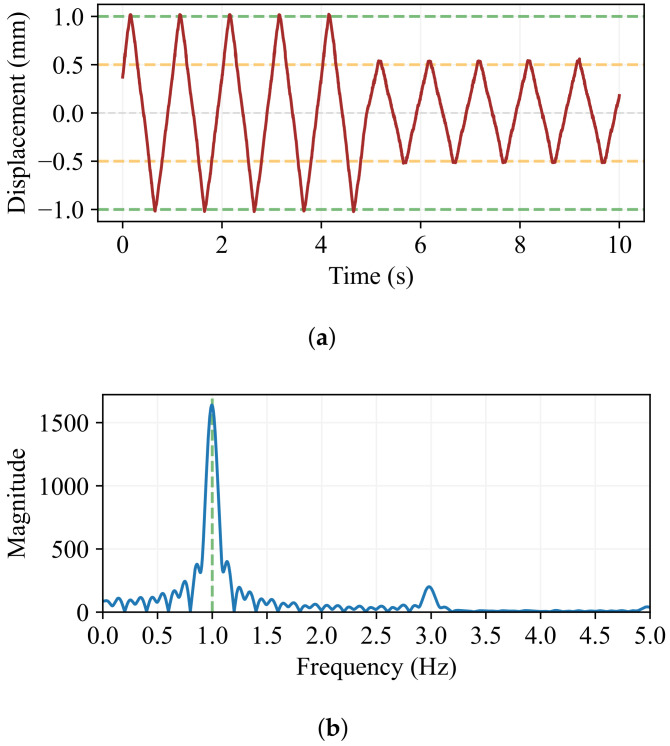
The amplitude change test of a 1.0 Hz oscillation, starting with $A_{i}=1.0$ mm and changing to $A_{f}=0.5$ mm. (**a**) Amplitude evolution over time. The change is visible around 5 s. (**b**) The frequency spectrum. The central frequency is clearly not modified by the amplitude change.

To check that real-time amplitude changes take place at the equilibrium point, an f=1.0 Hz oscillation was recorded for 10 s, changing the amplitude from $A_{i}=1.0$ mm to $A_{f}=0.5$ mm. As expected, the amplitude change occurs at the equilibrium point at around 5 s, as can be seen in Figure 10a, as well as in the frequency spectrum in Figure 10b, which shows that the oscillation frequency is not altered by the amplitude change.

### 4.4. Frequency Change

**Figure 11:**
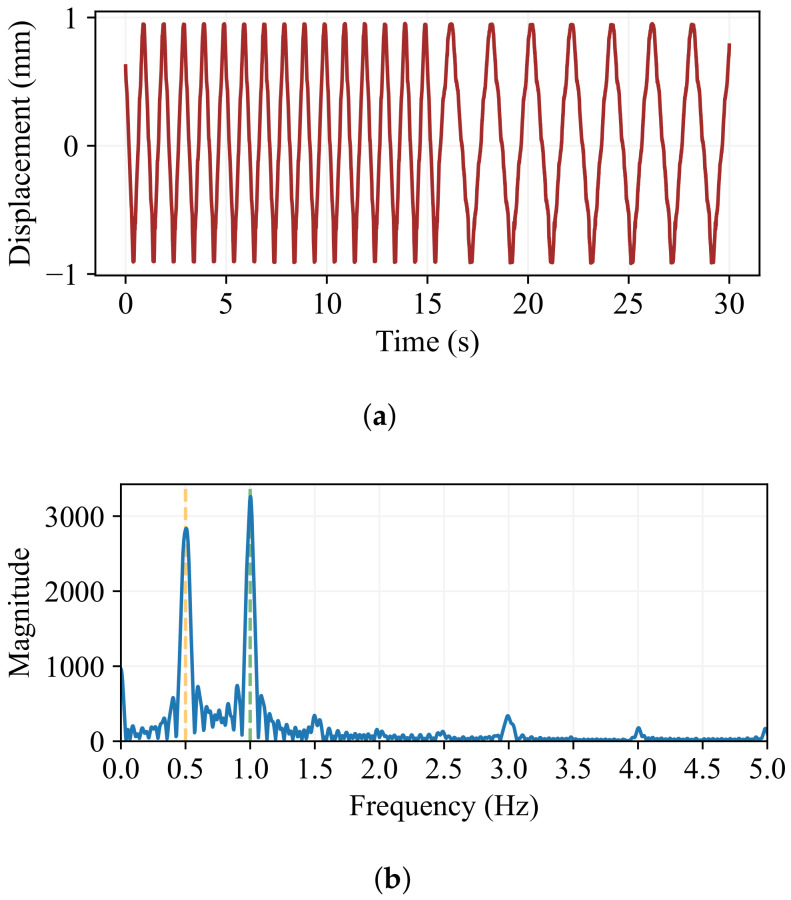
The frequency change of a 1.0 mm peak amplitude oscillation starting with $f_{i}=1.0$ Hz and changing to $f_{f}=0.5$ Hz. (**a**) Amplitude evolution over time. The frequency change is visible at around 15 s, and it does not alter the amplitude. (**b**) The frequency spectrum. Both frequencies are clearly detected.

Like amplitude changes, real-time frequency changes must happen when the oscillation is at its equilibrium point. A 30 s recording of an oscillation with A=1.0 mm going from $f_{i}=1.0$ Hz to $f_{f}=0.5$ Hz is shown in Figure 11a. At around 15 s of recording, the frequency change is visible, and it takes place at the equilibrium point. Its spectrum in Figure 11b confirms that the frequency changed between the mentioned values.

### 4.5. Portability

The system—both the microcontroller and the stepper motor—can be made portable via a 5V/2A power bank. The DRV8255 does not work with this configuration, but it can be substituted by the A4988, which has a lower resolution, as only 16 microsteps are available. The VREF value of the A4988 was set to ∼89 mV. Similar amplitude and frequency tests were carried out, and their results can be seen in Table 1 and Table 2, which show that the system is fully usable in this configuration and still meets the requirements for its application. Note that only a few values are shown in these tables for readability.

## 5. mmWave Radar Performance

Once the mechanical oscillator had been tested and calibrated using the OptiTrack system, amplitude and frequency tests were repeated using a mmWave radar. These studies aimed to verify the correct configuration of a mmWave radar to measure sub-millimetre oscillations precisely.

**Figure 12:**
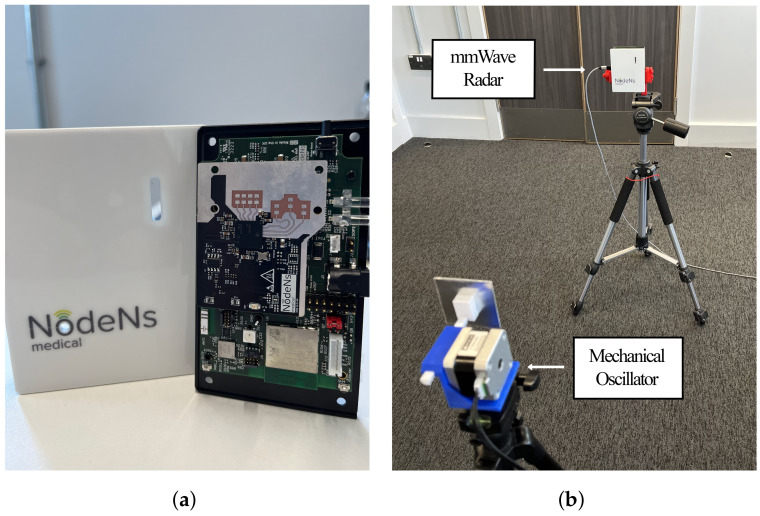
Radar hardware and system setup for testing. (**a**) NodeNs IWR6843ISK-based mmWave radar. (**b**) mmWave radar and mechanical oscillator mounted on tripods.

The mmWave radar used in these experiments is an FMCW radar manufactured by NodeNs Medical Ltd., (London, UK), based on Texas Instruments’ IWR6843ISK chip [31], which can be seen in Figure 12a. The device has been configured using the parameters in Table 3 following the usual configurations seen in the literature. For all tests carried out, the 3D-printed rack was attached to a small aluminium sheet so that it would be easily detected by the radar. The motor and the mmWave radar were separated by ∼1 m and mounted on vertically aligned tripods, as seen in Figure 12b. This distance is comfortably located in the far-field region, which is given by $D\geq 2L^{2}/\lambda =1.09$ mm, with L=1.65 mm being the length of the antenna and λ=4.98 m the wavelength of the radar wave, calculated using the parameters in Table 3.

We repeated the studies from Section 4.1 and Section 4.2 with the mmWave radar and with a collection time of 12 s. As mentioned in Section 3.3, the sampling frequency is the inverse of frame periodicity, which, in this case, is $f_{s}=16.67$ Hz, enough to satisfy the Nyquist–Shannon sampling theorem.

**Figure 13:**
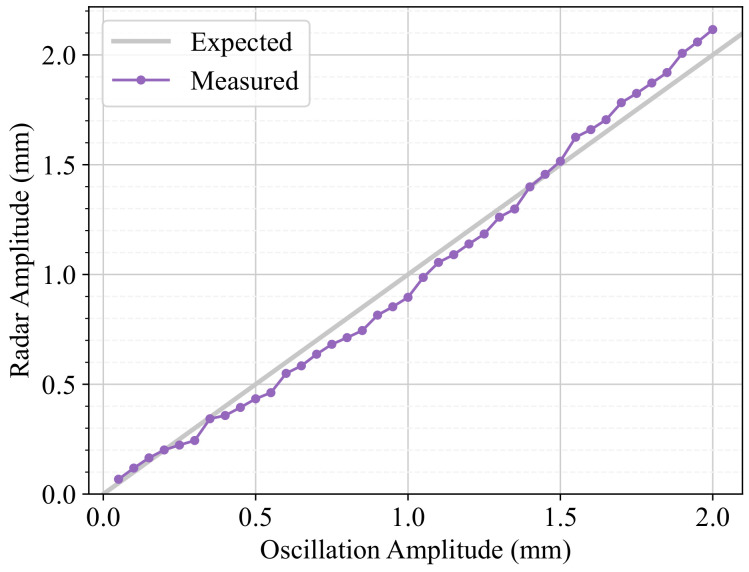
Amplitude study results with mmWave radar. The amplitude measured using the radar is plotted against the amplitude set in the device.

Figure 13 shows that the mmWave radar is capable of measuring the sub-millimetre oscillations with a peak amplitude MAE of 0.06 mm and a mean relative error of 7.88%. The greatest relative error is found for the smallest peak amplitude because the magnitude of the amplitude is very small, even though the absolute error is also small. There is a strong correlation between the measured and expected amplitudes, as shown by the close-to-1 slope of the regression described by the parameters in Table 4 and its sample Pearson correlation coefficient of 0.9964.

**Figure 14:**
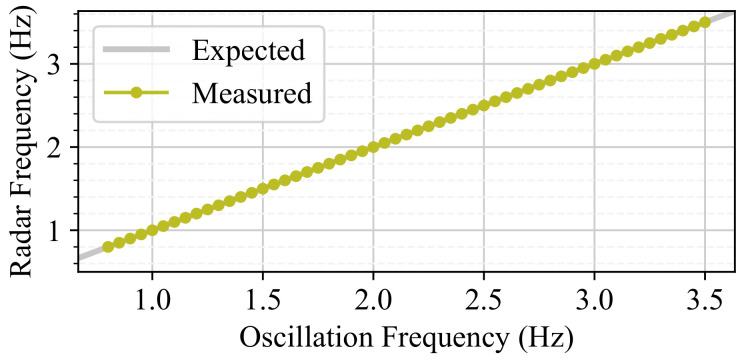
Frequency study results with mmWave radar. The frequency measured using the radar is plotted against the frequency set in the device.

Regarding frequency detection, it can be seen in Figure 14 that the mmWave radar could measure all of the oscillation frequencies tested, obtaining a 0% relative error computed with four decimal places. These results were obtained using an FFT, and the highest magnitude peak was selected as the detected frequency.

From these experiments, it can be concluded that the mmWave radar is properly configured to measure sub-millimetre oscillations with peak amplitudes and frequencies in the range of those found in chest movement produced by heart rate.

## 6. Conclusions

A mechanical oscillator has been proposed to test the configuration of a mmWave radar for precise sub-millimetre oscillation detection. First, it was tested and calibrated using a precise sub-millimetre motion capture system in the frequency range of [0.80,3.50] Hz and peak amplitude range of [0.05,2.00] mm, obtaining a 0.02% relative error for the oscillation frequency and a maximum peak amplitude relative error of 6%. Then, it was used to configure a 60 GHz mmWave radar to measure sub-millimetre oscillations, obtaining a 0% relative error for oscillation frequency detection and a mean absolute error of 0.06 mm for peak amplitude measurements. Thus, it has proven to be a reliable, wirelessly controlled mechanical oscillator with a fully controllable peak amplitude and frequency that researchers can use to configure mmWave radar devices prior to performing data collection with patients.

## Figures and Tables

**Table 1:** Amplitude calibration results while powering the microcontroller and motor via a 5V/2A USB power bank.

Expected (mm)	Measured (mm)	Rel. Error (%)
0.0500	0.0480	4.0714
0.1000	0.1052	5.2153
0.5000	0.4868	2.6459
1.0000	1.0047	0.4662
1.5000	1.4719	1.8761
2.0000	1.9838	0.8084

**Table 2:** Frequency test results while powering the microcontroller and motor via a 5V/2A power bank.

Expected (Hz)	Measured (Hz)
0.8000	0.7998
1.5000	1.4997
2.5000	2.4995
3.5000	3.4993

**Table 3:** List of configuration parameters for the NodeNs IWR6843-based mmWave radar.

Frequency	60.25 GHz	ADC samples	128
Idle time	7 μs	Sampling rate	4000 ksps
ADC start time	6 μs	Number of loops	32
Ramp time	50 μs	Frame periodicity	60 ms
TX start time	1 μs	TX antennas	2
Slope	30 MHz/μs	RX antennas	4

**Table 4:** The list of parameters for the regression between mmWave-radar-measured amplitude and oscillation amplitude.

Parameter	Value	Standard Error
Slope	1.0661	0.0148
Intercept (mm)	−0.0817	0.0174

## Data Availability

The raw data supporting the conclusions of this article will be made available by the authors on request.

## Appendix A

The 3D model of the rack-and-pinion system designed for this work is publicly available to download at https://www.thingiverse.com/thing:6530349 (accessed on 21 November 2024), where printing and refining instructions can also be found. In addition, the C code to program the ATSAMW25 SoC and the Python script to wirelessly control the device are available in https://github.com/felipeparralejo/mechanical-oscillator (accessed on 21 November 2024). It may work with other microcontrollers, but changes might be necessary.
